# “We Don't Have Any Clue What Will Happen to Them”: Perspectives of Women Who Had Gestational Diabetes About Long-Term Child Outcomes

**DOI:** 10.1155/jdr/6543458

**Published:** 2024-12-13

**Authors:** Oluwatoyin I. Oladimeji, Phyllis Ohene-Agyei, Luling Lin, Nike Franke, Jenny Rogers, Caroline A. Crowther, Jane E. Harding

**Affiliations:** Liggins Institute, University of Auckland, Grafton 1142, Auckland, New Zealand

## Abstract

In utero exposure to gestational diabetes mellitus (GDM) is associated with adverse long-term outcomes. Little is known about how mothers perceive these outcomes and the support they need for optimal outcomes for their children. We aimed to explore how women perceive the risk of adverse outcomes for their children exposed to GDM and the support they require for their optimal health. We conducted semistructured interviews with women who experienced GDM in at least one previous pregnancy. Data collection continued until saturation, and analysis followed an iterative thematic approach. Twenty-five mothers participated, and their perceptions about later outcomes for children exposed to GDM varied. Five themes were identified: relating GDM to the offspring's later health; reactions to the potential for poor later outcomes; impact on child growth, development, and behavior; maintaining optimal health from childhood to adulthood; and recommendations for long-term care. Most mothers received no information about potential later child outcomes; some based their views on assumptions. Some mothers who believed their children were at increased risk of poor outcomes expressed fear and worry, while others proactively ensured their children engaged in healthy lifestyle choices. Mothers emphasized the need for support within health facilities (information provision, linking antenatal with child records, and risk assessment) and in the community (social groups, home visits) to ensure optimal health of their children. These findings have potential implications for policy and practice changes to optimize later health outcomes for children exposed to GDM.

## 1. Introduction

Gestational diabetes mellitus (GDM) is defined as hyperglycemia first recognized during pregnancy, with glucose values above the normal ranges but lower than those diagnostic of overt diabetes [1]. It is a disorder of global public health importance, complicating millions of pregnancies globally [2]. In line with global trends, there has been a significant increase in the burden of GDM in New Zealand over the last three decades, with data from the National Women's Health, Auckland, which provides regional and national antenatal and postpartum maternity services, showing a dramatic increase in prevalence from 1.9% to 13.9% between 1990 and 2022 [3].

GDM not only impacts maternal health outcomes but is also associated with poor short-term outcomes in her offspring [4]. For example, around the time of birth, infants with in utero exposure to maternal hyperglycemia are at an increased risk of hypoglycemia, hyperbilirubinemia, being born large for gestational age, and respiratory difficulties [4, 5]. They are also at increased risk of adverse long-term outcomes, with one suggested mechanism being epigenetic changes brought about by exposure to aberrant intrauterine environment during fetal life [5]. Children whose mothers had GDM are at increased risk of adverse cardio-metabolic outcomes including increased adiposity, impaired glucose regulation, and insulin resistance, as well as allergies and poor neurodevelopmental outcomes [6–9]. GDM can perpetuate a vicious cycle of adverse intergenerational cardio-metabolic outcomes [10]. For example, a qualitative study on the experiences of GDM among Māori women living in Northland, New Zealand, revealed that most of them not only had a family history of diabetes but also had offspring who had developed abnormal glucose metabolism (GDM or Type 2 diabetes) during adolescence or young adulthood [10]. Despite these risks, many guidelines across the globe on the care of women who experience GDM and their offspring do not have clear guidance on measures to prevent these poor outcomes or how to monitor children exposed to GDM during fetal life [11–13].

Maternal perception about later health outcomes of their children born after pregnancies complicated by GDM may be an important step in breaking the cycle of poor intergenerational outcomes. Maternal perception of future child health risks may help shape how children are raised with respect to healthy lifestyle choices [14], which have been shown to be effective in preventing adverse cardio-metabolic outcomes [15]. However, there is limited evidence about how mothers who had GDM perceive future health risks for their children, how to maintain good health for their children, and the support they require to achieve this. Hence, we aimed to understand how women who experienced GDM view the risk of later adverse health outcomes in their children, their perception of the current routine well-child checks to meet their children's health needs, and what support they require to ensure their children maintain optimal health as they grow.

## 2. Materials and Methods

### 2.1. Study Design

We used a qualitative descriptive approach because it is a minimally theorized method helpful for understanding peoples' views and concerns about issues, thus providing insight of relevance to policymakers and practitioners [16]. We report our findings using the consolidated criteria for reporting qualitative research framework [17].

### 2.2. Ethical Consideration and Informed Consent

Ethical approval for this study was obtained from the Auckland Health Research Ethics Committee (AH26383), and written informed consent was obtained from participants.

### 2.3. Recruitment

Purposive sampling was used to recruit women who had experienced GDM in at least one previous pregnancy. Eligible women had participated in the Gestational Diabetes Mellitus Study of Diagnostic Thresholds (GEMS) trial and follow-up study, which were designed to determine which GDM diagnostic thresholds are associated with better short and long-term maternal and child outcomes [18]. Mothers were invited to participate in this study 4–7 years after the GEMS trial birth. Those who had indicated at the time of the follow-up study their willingness to be contacted for further research in the future were invited to participate via email. If there was no response after 2 weeks, a research team member (O.I.O.) telephoned them to confirm they had received the invitation and discuss their participation in the study. To help ensure a broad sample, invited participants included women from five major ethnic groups in New Zealand (Māori, Pacific, European, Asian, and MELAA [Middle Eastern, Latin American, and African]) [19] and women who did and did not have a child who had developed an adverse outcome (growth, neurodevelopmental, and atopic abnormalities identified during the GEMS follow-up study). Participant recruitment continued until data saturation was achieved.

### 2.4. Data Collection

An interview guide with semistructured open-ended questions (supplementary material (available here)) was used to guide the discussions. Pilot interviews were conducted with two women, and their feedback was incorporated into the interview guide and data collection process before the commencement of the study.

Two female researchers (O.I.O. and L.L.) conducted the semistructured interviews. Most interviews were conducted in English by O.I.O., a medical doctor with a Paediatric Fellowship qualification and formal training in qualitative research methods. L.L., a Chinese research fellow fluent in both English and Mandarin with experience in qualitative research methods, conducted the interview in Mandarin for a Chinese participant who requested the interview be carried out in her native language.

Each participant was offered the option to take part in the interview either online or face to face, as they preferred to ensure the voices of busy mothers who may not have the time to attend in-person sessions were heard. In-person interviews were conducted in a quiet room at the Clinical Research Unit of the Liggins Institute, University of Auckland, and were audio-recorded. Online interviews were conducted via Zoom and were audio and video recorded. Interviews lasted an average of 40 min and were transcribed verbatim by O.I.O. Participants had the opportunity to review their interview transcripts before they were analyzed.

### 2.5. Data Analysis

**Figure 1:**
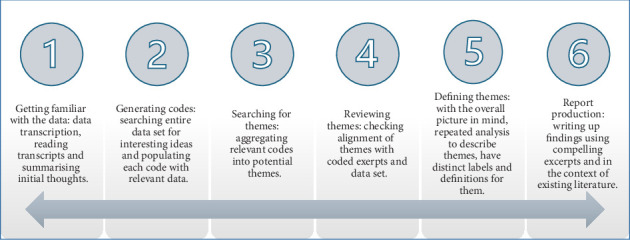
Phases of thematic analysis [21].

Interview transcripts were imported into NVivo v.14 [20], and data analysis was conducted in six multidirectional stages using iterative thematic analysis [21] (Figure 1). Two researchers (O.I.O. and P.O.-A.) independently coded the data and agreed after the initial phases on the coding template to use for data analysis. Themes were generated iteratively using an inductive lens as they were not predetermined but flowed from the data. Pseudonyms are reported instead of real names to protect the identities of participants and their children.

### 2.6. Reliability and Validity

We took several measures to ensure our study findings were reliable and valid [22]. First, we recruited participants from a wide range of ethnic backgrounds to strengthen the transferability of our findings. Second, we gave participants the opportunity to be interviewed in their native language and review the transcripts to enhance accuracy and credibility. Third, two reviewers independently did the coding, and themes were reviewed by all authors to reduce independent reviewer bias. Fourth, we sought out similarities and differences through the data set so that different perspectives from participants were reported. Lastly, we supported our findings with thick and rich verbatim narratives from the participants.

## 3. Results

Data were collected between September and December 2023 until data saturation was attained. Sixty-five mothers were invited to participate, of whom 26 did not respond to any form of contact. Of the 39 mothers who responded, 25 participated, 9 were interested but could not be reached after initial contact, and 5 declined to participate because they were busy. Of the 25 participating women, 6 (24%) were European, 5 (20%) were Māori, 2 (8%) were Pacific, 3 (12%) were MELAA, and 9 (36%) were Asian of whom four (44%) were Indian (Table 1). Twenty-three (92%) women opted for online interviews, while 2/25 (8%) were interviewed in person. Six (24%) women also volunteered that they had training to become health professionals, five (83%) of whom were currently practicing. Six (24%) of the women had experienced recurrent GDM in one or more pregnancies following the GEMS trial. We found five themes and 12 subthemes (Table 2). The themes were relating GDM to the offspring's later health; reactions to the potential for poor later outcomes; impact on child growth, development, and behavior; maintaining optimal health from childhood to adulthood; and recommendations for long-term care.

## 4. Relating GDM to the Offspring's Later Health

Participants had a wide range of perceptions about the risk of later adverse outcomes for their children. While some expressed uncertainty regarding any association between GDM and later outcomes, others believed there were no later risks, while still others believed their offspring had a higher chance of negative long-term outcomes.

Erm, because I do not know enough about the impact that this [exposure to GDM] has had on, you know, my children. (Sian, who had GDM in two pregnancies)

When you are saying that you wanna talk about what impact [later] it would have on my child, I do not think there is anything, nobody told me there were any potential negative outcomes [long-term] for him from me having gestational diabetes. (Susan)

So, I think, obviously, the long-term effect that you know it [in-utero exposure to gestational diabetes] has on a baby, or, you know,… obviously like it's, it's negative, it's not, it's not something that you want, and you know, erm it could be related to weights erm and yeah. (Rachel)

Perception of later risks was shaped by different factors including nonmodifiable (genetics) and modifiable (body size, upbringing, adopted lifestyle) factors. Women with a genetic predisposition related to race or family history believed their children had a higher chance of developing poor outcomes. Other mothers believed their children's risk of poor outcomes later in life depended more on the lifestyle choices they adopted than on whether they were exposed to GDM.

I do think they [children] are probably genetically prone to it [type 2 diabetes], they are Indians, they 100%, they are not mixed, so, it's not like I need to, I know they'll get it from both sides, because my husband's dad also has it, so they particularly they are prone to it. (Shammah, who had GDM in two pregnancies)

John, he's a very sports boy, like an exercise boy, if he keeps doing this, his life, he will not get the diabetes, I think. (Jane)

I do not think so [GDM may impact later child health], I think it's the way you raise them and stuff. So yeah, I do not think you could really blame it on you diabetes while you are pregnant, as long as you look after them and make sure their health is fine. (Esther)

For those who believed their children had higher chances of later poor outcomes, their views on the impact of GDM on potential later outcomes also varied. While most believed their offspring had a higher chance of developing Type 2 diabetes, others believed that in addition, their children were at an increased risk of other metabolic, cardiovascular, or neurodevelopmental disorders. Their views were driven by a variety of factors, including having some health-related training or working as health professionals. Two women (Asher and Rachel) who have training in different aspects of health and well-being believed later health risks for their offspring were not just limited to Type 2 diabetes but also included other metabolic and cardiovascular abnormalities such as overweight, obesity, hypercholesterolemia, and atherosclerosis.

I think she, I think she probably would get gestational diabetes, like I did, and then yes, from there she might [get diabetes], same as you know, I sort of think probably the same as me. (Mercy)

I did a lot of reading about, you know, what could happen if Mary got it … you know they talk about stuff, you know, like low mental development, or potentially being on the spectrum, and like so many, there are so many like effects. (Josephine)

I know that it [gestational diabetes] can have some metabolic effects on Isaac as well, you know he's got increased risk, risk of obesity and other metabolic disease. (Asher)

She would be more likely to develop type 2 diabetes, she would be at higher risk of, you know, having high cholesterol, erm and you know clogged arteries, and you know, being overweight or potentially obese, so yeah, I do believe that that could contribute to later, to her in later life. (Rachel)

Rachel also further explained the possible link between exposure to gestational diabetes and later child health. She stated that exposures from conception to the second birthday (first 1000 days after conception) could affect life-long health. Consequently, she expressed concern that she might be passing on certain factors, thereby increasing her daughter's risk of later adverse outcomes.

I know that basically when you are pregnant, all of your vitamins and nutrients, and everything is going towards that baby and I know how important erm you know, they say the first 1,000 days, and that's 1,000 days from conception. So, you know even the fact that they had to prick her foot after she was born so clearly, you know that they had concerns that she could potentially have high sugar levels, erm so for me, then that sort of you know showed me, oh okay, like, I can be passing things on. (Rachel)

Another factor influencing women's perceptions of later outcomes for their children was the extent of information available to them. More than half of mothers shared that they had never been informed about any potential risk of later outcomes for their offspring, leading some mothers to form their views on assumptions. For example, Roseline, a practicing health professional, explained it as having no clue about the potential outcomes.

I'm sort of thinking he [child] still has a chance to get diabetes, I do not know, I did not do that research, but I just assumed … Yeah, you know how they say for women, who have gestational diabetes after, there's number, I do not know the number, you can develop type 2 diabetes, for kids, for the children from those mothers, no information, no information about it, we do not have any clue what will happen to them … I do not have any clue about their fate, that when we are focussed about the, only women who had gestational diabetes, but not the children who are born from us (laughs). (Roseline)

For other women, perceptions of later child outcomes were formed by their experiences working as health professionals and personal research including using sources with varying degrees of reliable information like Google.

To be honest, it's probably through my job [health professional] rather than through any kind of health care information that I was given. (Asher)

I think again, it's because of that, you know, very quick, frantic research [on Google] that I did in the last trimester, the things that could stand out is, obviously like development and yeah, mental, or physical development in the child. (Josephine)

Other women explained that their antenatal health care provider counselled them on the risk of later adverse outcomes for their children. However, this was done only within the context of encouraging them to improve their lifestyle in pregnancy and not at a separate counselling session about potential later risks for children as mentioned by Jane.

Erm, because when I was diabetic when I was pregnant, I was found to be diabetic, the midwife said that if I do not control my diet or weight, after that delivery, John will get the diabetes more easier, get a diabetes more easier in in his future, so that's what I learnt from the midwife at that time. (Jane)

## 5. Reactions to the Potential for Poor Later Outcomes

Reactions to the knowledge of increased risk of possible adverse later outcomes were also mixed. While some women expressed negative emotions such as fear and worry, others were hopeful, proactive, inquisitive, or focused on risk reduction.

I'm fearful, I just do not want them [children] to get obese, or I do not want them to get type 2 diabetes, because I had gestational diabetes. (Jamie, who had GDM in three pregnancies)

I worry about my kids as well, I stop them from eating too much fruits at once, and I try to keep them active, cause I know they are at risk of getting diabetes later on in the life, yeah. (Lilian, who had GDM in two pregnancies)

We [mothers] also want to know if these children are different from the control group of children in any way. (Julia)

I just kind of look hard for the signs of, like really being thirsty is like one of the signs that I look for, and neither of these boys are thirsty, I have to force water down it's just, erm so I just kind of look out for those signs, and I know to escalate it as needed. (Shammah, who had GDM in two pregnancies)

### 5.1. Impact on Child Growth, Development, and Behavior

Some mothers reported that those children whose pregnancies were complicated by GDM were bigger and had bigger appetites compared to their other children. Other mothers, including those who were health professionals, had observed neurodevelopmental and allergy-related abnormalities in their children but were uncertain if these were related to in utero exposure to GDM.

She's definitely my chubbiest little baby (laughing), and also insane that she actually is the one who eats the best … her speech is a lot slower compared to the rest [child's other siblings], and I kind of imagined her to be faster, because there was more people talking around here all the time, older children, erm, she's at school, but she still even has trouble, there's nothing wrong with the hearing, but she does have trouble pronouncing words correctly still, so, yeah … like when I do see things with her, I kinda often do think back to whether it [observed differences] was that [exposure to GDM] that causes her to be that way or not, you know… I do not know if it [observed differences] has nothing to do with that [exposure to maternal GDM], but these are the things I now notice at 5 that hold her back a wee bit. (Sue)

So, my mom's like erm, maybe we can send her to the doctor and assess her, but what I feel like is she can be, she can be hyper focused, because we are leaning, we are talking about ADHD, because she's so super active and yeah, she is hyper focused on the things that she likes…she usually gets distracted… I'm actually not sure [if ADHD is linked to maternal diabetes], I have not really kind of searched on that. (Ruth)

We recognize that he had asthma as well in life, I'm not sure if this is diabetes related. (Blessing)

### 5.2. Maintaining Optimal Health From Childhood Into Adulthood

Most mothers ensured their children were physically active and had a healthy diet. Some mentioned a conscious choice to expose their children to a healthy lifestyle early and thus create healthy patterns for them to engage in from childhood to adulthood.

We have already started with keeping him healthy as well, as I'm giving him healthy foods and stuff like that. So like, we are we, we believe that we started early it will stay with him, and he and it will always be, and he'll always enjoy eating that kind of food, so erm yeah, so we are start, to try to get started early for him to get his body prepared and ready, and everything for the future. (Caroline)

Mothers also ensured opportunities for healthy lifestyles like sports, limiting unhealthy food options, and actively teaching their children about developing a healthy lifestyle to prevent later poor outcomes.

Erm well, she participates in sports, and I try to ensure that she has a healthy diet, and I try to limit, you know, treats, you know, so that it's not every day. (Rachel)

When we go to the doctors, or when we go to the Indian stores or something, they will offer lollipops, or if they give it, and I just take it, and I hide it away, just chuck it away, because I know those things are full of sugar, and once he [son] takes that, and he's gonna always want to. Caroline)

I have been talking to her, I think, like talking to them while they growing up and stuff, you know, that exercise is important, and erm yeah, like eating wise and stuff, not too much carbs, I do talk to her,… I do talk about diabetes at home with my kids, because they sometimes eat sweets and stuff, and I do talk about diabetes that if you are doing this you might get diabetes. (Lilian, who had GDM in two pregnancies)

However, factors mothers identified as barriers to ensuring healthy lifestyles for their children were socio-economic, family-related (habits and parenting style), limited resources, organizational (school based), and personal (innate child features and limited knowledge of healthy dietary options).

I feel sad because you cannot, there's no microwave to heat up their food at school, so you have to do sandwiches with, like, you know, like lunch or ham, or something like that, so, and I'm also concerned about food poisoning. (Jamie, who had GDM in three pregnancies)

My son is autistic, he received the diagnosis of autism, so it's hard for me to feed him, he's a picker eater, but he does eat, I encourage him to eat the fruit and veggie, but it's very hard, err few food to eat, just eats broccoli, eats broccoli, and peach and blueberry, so and yeah, that's the thing he eats, the rest, he just eats carbohydrates, I said oh my gosh, what am I going to do, I'm aware, but it's hard for me. (Roseline)

Similarly, mothers voiced their concerns regarding not having much support for long-term care outside the existing routine options (well-child and general practice clinics), which are sometimes out of the reach of some families. They also highlighted the need for a tailored approach to the care of children exposed to GDM.

There does not seem to be much support with regard to the child, apart from, like the Plunket [well child] visits and if I go to the GP [general practitioner] with a concern, but I and just think about all the families that do not actually have access to a lot of that. (Sian, who had GDM in two pregnancies)

Well, I think the check-ups for the kids from birth to before school [routine well child visits] are general, so every child will have that, I think erm, here in New Zealand, but not every child has a mother who had gestational diabetes during pregnancy, so, I feel like there needs to be an extra element to follow up that specific detail. (Mandy)

One mother also highlighted that well-child clinics are not available for children older than 5 years, despite exposure to GDM potentially having an ongoing impact on a child's growth.

We need more support for the baby to see what's happening with their life afterwards, is the growth, the height and the weight is okay? So yeah, I think 5 years old is not good enough. (Blessing)

### 5.3. Recommendations for Long-Term Care

Mothers believe a lack of awareness of exposure to GDM by well-child providers could contribute to the lack of tailored care for their children. They highlighted the need for linking health records, using an algorithm to assess and classify health needs, and that care beyond the routine well-child checks should be available to ensure their children receive optimal care tailored to their needs.

Yeah, also for the kids as well, like for the Plunket [well-childcare providers], they should know, since, like you know, the linking of details and information, I guess they should be informed as well that hey, the mom got GDM, so you need to monitor more the kid for some signs and symptoms … yeah, that's right to see what happened afterwards, like after 5 years and see what the progress is. Is the baby born with the gestational diabetes healthy? Are there any other complications? So, I would suggest, yeah, if they have like a follow up on that. (Ruth)

Erm, there could be a section when they have it, they [well-child clinic nurses] could, because they have like a questionnaire, and every time we would go, and they would maybe in that questionnaire there could be something just ask, you know, do you have any, and then, if the answers, yes, they give you questions and follow up points, once it's in the system, then it shows. (Caroline)

One mother, a health practitioner, stressed the importance of providing balanced information that empowers mothers on measures to reduce the potential for poor outcomes in their offspring's future. Similarly, most mothers longed for more education and information about healthy options for their children so that they could maintain optimal health.

I mean, they should be, like I say, find balance between scaring them and informing them, but yeah, I think, you know, if there's research to say that their children are risk, they should be informed of that, and they should be informed of the strategies to help them mitigate that risk. (Asher)

The only thing that I say is to give them more information on diabetes, on what foods erm will trigger the diabetes [in children], or what is high in sugar, even if, because even like your fruits, you know, some of them have high content of sugar, some of them are low content of sugar, so even though you may think you are eating healthy, but you are eating something which has got lot of sugar in it, you know, so then again, it's just, I think, just educating moms. (Caroline)

The need for healthy lifestyle education provided to mothers and other family members during supportive home visits was also highlighted. One mother also suggested support groups for mothers whose benefits include providing children with peer support for establishing healthy lifestyle patterns.

Having someone support them like, you know, come and talk to them as a family,… I just feel that maybe if they had someone support person like for the family, and they come and visit and see how things are at home for them and the environment and stuff, yeah, just a bit more support around those things. (Lilian, who had GDM in two pregnancies)

When I first speak about a mom's group for a coffee chat group, already, I guess I think about the kids becoming friends (smiling), and then, like you know, like I think about, you know, you'd made up their lunch box, you know that open up their lunch boxes and eventually, when they are a bit older, and then yeah, like just adopting those healthy eating habits with like-minded mums and kids, I guess. Yeah, yeah. (Sian, who had GDM in two pregnancies)

## 6. Discussion

We have heard the voices of mothers who experienced GDM and found their perspectives about later health outcomes for their children varied based on prior knowledge of GDM and later child outcomes and awareness of the roles genetics or lifestyle choices play in lifelong health.

Some mothers believed that exposure to GDM does not impact later child outcomes, asserting instead that upbringing plays a more important role. This is at variance with existing literature highlighting the intergenerational poor outcomes associated with GDM [23, 24] and that exposure to GDM is associated with adverse long-term growth, developmental, metabolic, and cardiovascular outcomes [6–8, 25]. This perspective could be due to the lack of information from healthcare providers about the future health risks of children exposed to GDM. Most mothers in our study stated that they received no information from healthcare providers about the future health risks for their children, and many of them, including some health professionals, were uncertain about potential outcomes. Some mothers based their views on assumptions, and this has been previously reported to be associated with negative consequences since it is linked with not adequately addressing specific health issues, thus promoting poor outcomes [26]. Similar to our findings, it has been previously reported that parents were anxious and worried when they had little insight about exposures and potential later outcomes for their children [27], further reiterating the need to give parents balanced information not only on later risks but also on how to reduce potential adverse outcomes based on high-quality evidence. This would, however, call for more high-quality longitudinal studies that will provide such evidence. Although previous studies have shown clear benefits of improved lifestyle choices or metformin on preventing or delaying the onset of adverse cardiometabolic outcomes in women who experienced GDM [28–30], there is a dearth of evidence on the factors that would mitigate the later health risks associated with in utero exposure to GDM [24].

Similar to our findings, Neufeld [14] reported that mothers who believed their children exposed to GDM were at an increased risk of developing Type 2 diabetes were fearful and concerned about this possibility but also made conscious efforts to ensure these children were not exposed to unhealthy lifestyle choices. It is well established that healthy lifestyle choices improve health and well-being and reduce the risk of adverse metabolic and cardiovascular outcomes [15], and that incorporating increased physical activities and healthy dietary patterns is both effective and cost-effective in lowering body mass index *z* scores in school-aged children [31]. However, this is yet to be explored within the context of in utero exposure to GDM and lifelong health [24]. Studies not only focused on the theory and practice of lifestyle choices but also include cultural sensitivity and acceptability, which are needed to support optimal health and prevention of poor outcomes for children exposed to GDM [28, 32]. Such evidence would help to provide guidance to mothers in improving the life-long health of their children.

Consistent with views expressed by mothers in our study, McIntyre et al. [24] also reported that understanding what care is optimal in the long-term for children exposed to GDM is hindered by limited follow-up after birth, reducing opportunities for the prevention of poor intergenerational outcomes. Many GDM management guidelines outline the risks of adverse multisystem long-term maternal and child outcomes following GDM [11, 12, 25] and have specific guidance on measures to prevent and promptly detect poor later maternal outcomes but provide no recommendations about best practices to promptly detect adverse outcomes in their children. Most well-child clinics globally, including in New Zealand, provide routine vaccinations and monitor the growth and development of children for the first 4–5 years of life, but mothers in our study suggested the provision of more targeted care by health professionals to reduce the risk of poor outcomes for their children. This would require linking antenatal records with postbirth records, providing information on potential outcomes and how to prevent these, utilizing a risk assessment algorithm to predict outcomes, and follow-up even after the child is no longer within the age bracket for the routine well-child checks. Incorporating consumer views into clinical practice guidelines (CPGs) is increasingly recognized as necessary in providing consumer-friendly recommendations that positively impact outcomes [33]. Hence, potential opportunities for improved care for children exposed to GDM, as desired by mothers in our study, should be considered for incorporation into CPG for the long-term care of women who had GDM and their children.

Support outside the regular healthcare setting in the form of home visits and peer coffee groups was also highlighted as important for mothers to ensure long-term health and well-being for their children. A systematic review and meta-analysis of 21 high-quality randomized controlled trials examining the impact of home visits on childhood outcomes highlighted that home visits were effective for preventing child abuse, reducing cognitive and behavioral childhood problems, lowering the incidence of abnormal weight gain and health problems in childhood [34], some of which have been raised as key concerns by mothers in our study. The value of parent support groups aimed at improving healthy lifestyle practices and choices for children, as highlighted by some women in our study, has been previously reported [35, 36]. For example, Bektas et al. [35], in their qualitative study on utilizing parent–child meetings to support parents, highlighted that weekly community-based parent–child groups were associated with improved parental skills, knowledge, and practices around healthy lifestyle choices and behaviors for their children. This model would have resource implications but could be modified based on contextual factors to provide ongoing support for mother–child dyads exposed to GDM.

Our study has several strengths. To our knowledge, this is the first study specifically aimed at exploring how mothers perceive future health risks and needs for their children exposed to GDM. We used a qualitative descriptive methodology that is minimally theorized and stays close to participants' views, hence useful to provide insight relevant to health practitioners and policymakers [16]. Including participants from diverse ethnic backgrounds makes our findings transferable in New Zealand and potentially broader contexts. A limitation of our study is that we did not specifically include health professionals involved with the care of women with GDM and or their children to hear their perceptions on later outcomes and needs of children exposed to GDM. However, some of our study participants were health professionals involved in the care of women who had GDM and children.

## 7. Conclusions

Mothers had varied perceptions about later health risks for their children exposed to GDM. Many were uncertain about the links between concerns they had for their child and exposure to GDM. This was largely due to a lack of information about later outcomes from health professionals. Mothers desired more information, targeted follow-up, and support within and outside health facilities for the optimal care for their children, which has potential implications for policy and practice changes to prevent poor, long-term, and intergenerational outcomes associated with GDM. However, more high-quality studies are needed to provide clear guidance on best practices for the long-term care of children exposed to GDM in utero. Studies on antenatal and well-child providers' perception of later outcomes following in utero exposure to GDM and support for optimal health are also needed.

## Figures and Tables

**Table 1:** Study participants and methods preferred for interviews.

**Study ID**	**Mother's ethnicity**	**Child's age (years)**	**Online or in-person interview**
P01	European (New Zealand)	5	Online
P02	European (New Zealand)	6	In-person
P03	Asian (Chinese)	7	Online
P04	European (New Zealand)	7	Online
P05	Asian (Chinese)	7	Online
P06	Pacific (Samoan)	6	Online
P07	Māori	5	Online
P08	Asian (Filipino)	6	Online
P09	Asian (Filipino)	5	Online
P10	Māori	5	Online
P11	European (New Zealand)	6	Online
P12	Pacific (Tongan)	6	Online
P13	European (British)	6	Online
P14	European (British)	5	Online
P15	Asian (Sri Lankan)	6	Online
P16	MELAA (African)	4	Online
P17	Asian (Indian)	4	Online
P18	Asian (Indian)	4	Online
P19	Asian (Indian)	4	In-person
P20	Māori	4	Online
P21	MELAA (Middle Eastern)	4	Online
P22	Asian (Indian)	5	Online
P23	Māori	4	Online
P24	MELAA (African)	4	Online
P25	Māori	5	Online

**Table 2:** Themes, subthemes, and codes.

**Theme 1: Relating gestational diabetes mellitus to the offspring's later health**			
**Subthemes**	Risk of later poor outcomes	Nonmodifiable and modifiable factors influence risk	Information on later outcomes
Codes	− Increased risk of adverse outcomes (growth, metabolic, cardiovascular, and neurodevelopmental) − Exposures in the first 1000 days after conception impact life-long health − No long-term risks − Uncertain if any association exists	− Family history, genetics, and race − Body size − Adopted lifestyle − How kids are raised	− No information − Making assumptions − Varying sources (personal research, career or medical training, and antenatal health professionals)

**Theme 2: Reactions to the potential for poor later outcomes**			
**Subthemes**	**Negative**	**Positive**	
Codes	− Fear − Worry − Concern	− Curious − Hopeful − Proactive − Focusing on risk minimization	

**Theme 3: Impact on child growth, development, and behavior**			
**Subtheme**	**Growth, behavior, and development differences**		
Codes	− Size and appetite − Neurodevelopmental concerns − Uncertainty if observed features are linked with exposure to GDM		

**Theme 4: Maintaining optimal health from childhood into adulthood**			
**Subthemes**	**Healthy lifestyle to prevent poor outcomes**	**Barriers to healthy lifestyle**	**Well-child clinics**
Codes	− Physical activity and healthy diet − Hiding and chucking unhealthy food − Teaching children about the benefits of healthy lifestyle choices − Starting early to ingrain healthy lifestyle	− Cost of engagement in sports and healthy meals − Limited resources for healthy diet choices − No microwaves in schools − Differences in parenting styles − Increased screen time − Underlying medical problem	− Not much support − No tailored approach to care − No information on healthy dietary choices for kids − No follow-up after 5 years

**Theme 5: Recommendations for long-term care**			
**Subthemes**	**Improved sharing of relevant risks and outcomes**	**Care received**	**Social support**
Codes	− Linking health information so care providers are aware to provide tailored care − Balanced information on later outcomes to mitigate risk but not induce fear − Provide information on healthy dietary options	− Risk assessment for individualized follow-up − Follow-up after 5 years	− Home visits to follow up on healthy lifestyle − Mum's support groups that benefit kids

## Data Availability

The data for this study will be shared with researchers who have a sound proposal and upon reasonable requests. Requests to access data should be sent to the Data Access Committee at researchhub@auckland.ac.nz.
