# Supplementary material for: “We Don't Have Any Clue What Will Happen to Them”: Perspectives of Women Who Had Gestational Diabetes About Long-Term Child Outcomes
Source: J Diabetes Res. 2024 Dec 13;2024:6543458. doi: 10.1155/jdr/6543458 (PMC11661868; doi:10.1155/jdr/6543458)
Supplement: Supporting Information — Additional supporting information can be found online in the Supporting Information section. Interview guide. [file 6543458.f1.docx]

Interview guide.

1. What are your thoughts about what experiencing diabetes when you were pregnant with [child’s name] could mean for his/her health now or even later in the future?

Prompts

- What could having experienced diabetes during [child’s name] pregnancy mean for his/her growth and development?
- I would also like to know your thoughts about health issues that may occur more frequently in children whose mothers had GDM when compared to children whose mothers’ blood glucose levels remained normal throughout pregnancy?
- I am interested in your thoughts about [child’s name] risk of health problems later in the future.
- How did you learn about these?

1. What are your views on things that can be helpful for [child’s name] or other children whose mothers had diabetes to ensure they maintain good health as they grow up, months and even years after birth?

Prompts

- What are some things that can be done for/to children whose mothers had diabetes during their pregnancy so they can be healthy while growing up?
- How did you learn about these?

1. In New Zealand, there are routine healthy checks for children, from six weeks to the before-school check; what are your thoughts about if these checks are adequate for [child’s name] or other children whose mothers had diabetes during pregnancy?

Prompt

- What specific thing would you like to see included in their care, and why?

1. What support do you think would be good for children whose mothers had diabetes during pregnancy to ensure they maintain good health as they grow?

Prompt

- Is there any other thing that you think [child’s name] or other children whose mothers had diabetes during pregnancy may benefit from?
